# Intraocular Lens Power Calculation after Small Incision Lenticule Extraction

**DOI:** 10.1038/s41598-020-63118-0

**Published:** 2020-04-06

**Authors:** Nikolaus Luft, Jakob Siedlecki, Benedikt Schworm, Thomas C. Kreutzer, Wolfgang J. Mayer, Siegfried G. Priglinger, Martin Dirisamer

**Affiliations:** 1 University Eye Hospital, Ludwig-Maximilians-University, Munich, Germany; 2 SMILE Eyes Clinic, Linz, Austria

**Keywords:** Outcomes research, Therapeutics

## Abstract

With more than 1.5 million Small Incision Lenticule Extraction (SMILE) procedures having already been performed worldwide in an ageing population, intraocular lens (IOL) power calculation in post-SMILE eyes will inevitably become a common challenge for ophthalmologists. Since no refractive outcomes of cataract surgery following SMILE have been published, there is a lack of empirical data for optimizing IOL power calculation. Using the ray tracing as the standard of reference – a purely physical method that obviates the need for any empirical optimization - we analyzed the agreement of various IOL power calculation formulas derived from the American Society of Cataract and Refractive Surgeons (ASCRS) post-keratorefractive surgery online calculator. In our study of 88 post-SMILE eyes, the Masket formula showed the smallest mean prediction error [−0.36 ± 0.32 diopters (D)] and median absolute error (0.33D) and yielded the largest percentage of eyes within ±0.50D (70%) in reference to ray tracing. Non-inferior refractive prediction errors and ±0.50D accuracies were achieved by the Barrett True K, Barrett True K No History and the Potvin-Hill formula. Use of these formulas in conjunction with ray tracing is recommended until sufficient data for empirical optimization of IOL power calculation after SMILE is available.

## Introduction

Since its commercialization in 2011, small incision lenticule extraction (SMILE) has become an integral part of modern keratorefractive surgery. To date, however, research has failed to address an anticipated issue after SMILE that is inherently tied to any kind of keratorefractive procedure – future intraocular lens (IOL) power calculation.

History has taught us that IOL power calculation following excimer-based photoablative procedures [laser *in situ* keratomileusis (LASIK) or photorefractive keratotomy (PRK)] is complicated by three major issues^1^. First and foremost, as the natural anterior/posterior corneal curvature ratio is altered by keratorefractive surgery, topography (that only measures the anterior and extrapolates the posterior curvature) is flawed resulting in overestimation of corneal power readings (index of refraction error). In addition, many standard IOL calculation formulas utilize the measured corneal power as part of the estimation of the postoperative axial position of the IOL (formula error). Moreover, keratometers are prone to overestimating corneal power as the central zone of effective corneal power (that had been artificially flattened by keratorefractive surgery) is not measured (instrument error)^1^. These factors concordantly predispose to unpleasant hyperopic refractive error after IOL implantation in eyes with prior myopic keratorefractive surgery.

Several methods have been introduced to minimize these refractive surprises after cataract surgery in post-LASIK/PRK patients, who commonly harbor high visual and refractive expectations. The clinical history method that takes into account the pre- and postoperative subjective refraction had been considered the gold standard for years^2^. Thereafter, a range of more sophisticated IOL power calculation formulas with superior accuracy was developed based on empirical optimization: some of which take into account pre-keratorefractive surgery data (e.g. the Masket^3^ or Barrett True-K formula), some do not incorporate any preoperative values (e.g. Shammas^4^ or Haigis-L formula^5^). Conveniently, a range of these formulas is readily accessible in the American Society of Cataract and Refractive Surgeons (ASCRS) post-keratorefractive surgery IOL power online calculator. Avoiding any empirical optimization, ray tracing represents a purely physical approach for IOL power calculation particularly attractive in eyes with previous LASIK or PRK^6–10^. This method involves direct measurement of the posterior corneal curvature (e.g. with Scheimpflug tomography) and obviates the need for historical clinical data.

With more than 1.5 million SMILE procedures having already been performed worldwide in an ageing population, IOL power calculation in post-SMILE eyes will inevitably become a common challenge for ophthalmologists. The dilemma we face when encountering the first post-SMILE patient requiring cataract removal with IOL implantation lies in the fact that, as of today, no single case of cataract surgery following SMILE has been published in the peer-reviewed literature. Hence, in the absence of sufficient clinical data for empirical optimization of our IOL power calculation, we must either utilize a physical approach (i.e. ray tracing) or rely on the aforementioned formulas optimized for Excimer-based photoablative procedures. The validity of these formulas in post-SMILE eyes seems questionable, as SMILE produces significantly different corneal shape changes as compared with fs-LASIK. In their study of 884 eyes having undergone either fs-LASIK or SMILE, Gyldenkerne et al.^11^ showed that the anterior corneal surface in the central 2.00 mm zone is steeper in post-SMILE corneas but flatter in the corneal periphery as compared with fs-LASIK, thus better preserving the natural asphericity of the cornea.

In the present study, we set out to compare the refractive prediction error of IOL power calculation in post-SMILE eyes between ray tracing and various empirically optimized formulas available in the ASCRS post-keratorefractive surgery IOL power online calculator. The results of this study may provide clinicians with guidance for their first IOL power calculations in cataractous post-SMILE eyes.

## Results

A total of 88 eyes of 88 patients [44 (50%) females] were included with a mean follow up after SMILE of 11 ± 12 months (range 3 to 54). Subjects’ baseline characteristics are summarized in Table 1. Mean IOL power as calculated by ray tracing was 20.91 ± 1.71D (range 16.50 to 24.50) with a predicted residual refraction of −0.01 ± 0.10D (range −0.16 to 0.17) of manifest refraction spherical equivalent.

**Table 1:** Subjects’ characteristics.

Parameter	Mean	SD	Range
Age (years)	34.1	8.6	20 to 63
Preoperative Manifest Refraction (D)			
Sphere	−4.01	1.82	−0.25 to −8.50
Cylinder	−1.05	1.07	0.00 to −5.50
Spherical Equivalent	−4.53	1.82	−1.50 to −8.75
Postperative Manifest Refraction (D)			
Sphere	0.11	0.43	−1.50 to 1.25
Cylinder	−0.38	0.29	0.00 to −1.75
Spherical Equivalent	−0.08	0.41	−1.88 to 0.75
SIRC (D of MRSE)	−4.45	1.78	−1.25 to −8.63
Axial length (mm)	24.94	0.98	22.76 to 27.32
ACD (mm)	3.69	0.29	3.14 to 4.73
Keratometry [mm (D)]			
K flat	8.44 (40.06)	0.40 (1.86)	7.68 to 9.31 (36.25 to 43.95)
K steep	8.27 (40.87)	0.37 (1.83)	7.55 to 9.25 (36.49 to 44.70)
K mean	8.36 (40.46)	0.39 (1.85)	7.62 to 9.28 (36.37 to 44.32)
White-to-white (mm)	12.3	0.4	11.5 to 13.2

SD, standard deviation; D, diopter; SIRC, surgically induced refractive change; MRSE, manifest refraction spherical equivalent.

**Figure 1:**
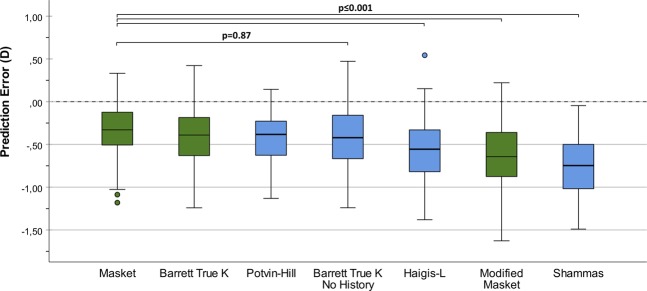
Prediction errors of IOL power calculation formulas as compared with ray tracing. Formulas were sorted by magnitude of mean error. Green boxplots show formulas that incorporate clinical history data and blue boxplots show formulas that do not use any prior-keratorefractive surgery data. The p-values of Tukey post hoc testing for pairwise comparisons between formulas are shown above the boxplots. (D, diopter).

The performance of the investigated IOL power calculation formulas in reference to physical ray tracing is summarized in Table 2 and boxplots comparing their prediction errors with ray tracing are presented in Fig. 1. On average, the formulas concordantly overestimated IOL power as compared with ray tracing. Of all investigated formulas, the Masket formula yielded the lowest ME (−0.36 ± 0.32D, range −1.18 to 0.33). However, ANOVA with Tukey post hoc testing revealed that the Barrett True K (p = 0.97), Barrett True K No History (p = 0.87) as well as the Potvin-Hill (p = 0.97) formula showed prediction errors on par with the Masket formula. The Haigis-L (p = 0.001), Modified Masket (p < 0.001) and Shammas (p < 0.001) formula showed statistically significantly larger prediction errors as compared with the Masket formula. The Shammas formula resulted in the largest IOL power overestimation with a ME of −0.75 ± 0.33D (range −1.49 to −0.05).

**Table 2:** Formula performance in comparison with ray tracing.

Formula		Prediction Error (D)			Absolute Error (D)		% of Eyes Within PE Range Indicated			
		Mean	SD	Range	Mean	Median	±0.25D	± 0.50D	± 0.75D	± 1.00D
Using prior data	Barrett True K	−0.40	0.33	−1.24 to 0.42	0.44	0.40	28	63	84	95
	Masket	−0.36	0.32	−1.18 to 0.33	0.39	0.33	34	70	91	95
	Modified Masket	−0.64	0.38	−1.63 to 0.22	0.65	0.64	17	36	63	83
Using no prior data	Barrett True K No History	−0.42	0.32	−1.24 to 0.47	0.44	0.44	28	57	86	97
	Haigis-L	−0.56	0.36	−1.38 to 0.54	0.58	0.56	18	43	65	90
	Potvin-Hill	−0.40	0.29	−1.13 to 0.14	0.42	0.38	27	64	90	98
	Shammas	−0.75	0.33	−1.49 to −0.05	0.75	0.75	8	25	50	73

D, diopters; PE, prediction error; SD, standard deviation; AE absolute error.

Concordantly, the Friedmann test revealed statistically significant differences between the absolute prediction errors (p < 0.001). Of the various tested formulas, the Masket formula achieved the smallest MedAE (0.33D) and MAE (0.39D). Post hoc analysis using Wilcoxon signed-rank pairwise comparisons with Bonferroni correction showed that the Barrett True K (p = 0.51), Barrett True K No History (p = 0.22) as well as the Potvin-Hill (p = 0.99) formula resulted in absolute prediction errors non-inferior to the Masket formula. In contrast, the Haigis-L, Modified Masket and Shammas formula (all with p < 0.001) showed statistically significantly larger absolute prediction errors.

**Figure 2:**
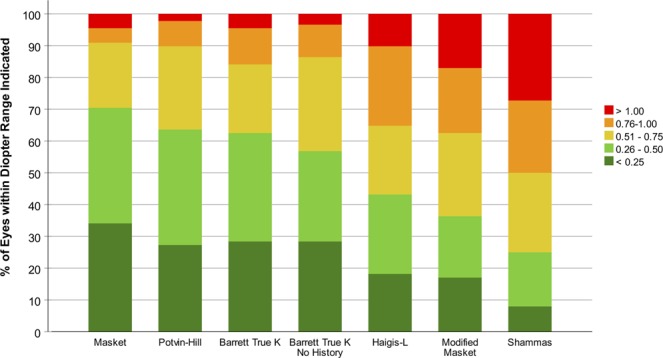
Stacked histogram analysis comparing the percentage of eyes within given prediction error ranges as compared with ray tracing. The formulas were sorted by the proportion of eyes within ±0.50D in descending order.

With 70%, the Masket formula yielded the highest percentage of eyes within a refractive prediction error of ±0.50D (Fig. 2). Fisher’s exact test with Bonferroni correction showed that the Barrett True K (p = 0.99), Barrett True K No History (p = 0.48) as well as the Potvin-Hill (p = 0.99) formula did not exhibit statistically significantly inferior ±0.50D accuracy whereas the Haigis-L (p = 0.002), Modified Masket (p < 0.001) and Shammas (p < 0.001) formula performed inferiorly in this regard. The Barrett True K no history method achieved the highest ±1.00D accuracy (98%) with all other formulas except the Modified Masket (p = 0.008) and the Shammas (p < 0.001) showing non-inferiority with p-values ranging from 0.34 (Haigis-L) to 1.00 (Barrett True K No History).

## Discussion

In the present work, ray tracing was considered the benchmark to compare the agreement of seven formulas available in the ASCRS post-keratorefractive surgery IOL power online calculator in post-SMILE eyes. The ray tracing method represents an alternative to post-keratorefractive surgery IOL power calculation formulas that include “fudge factors” (or “correction coefficients”), since this purely physical approach obviates the need for any empirical optimization and does not require any preoperative data. Hence, this technique cannot be flawed by postoperative refractive changes (e.g. owing to a maturing cataractous lens), a problem that is an inherent issue with all formulas that incorporate preoperative clinical history. We acknowledge the fact that analyzing actual postoperative refractive outcomes of cataract surgery after SMILE would be the gold standard to compare different IOL power calculation formulae. As of today, however, these clinical data are lacking. Hence, considering that ray tracing has been proven to be highly efficacious in estimating corneal power changes^9^ and calculating IOL power after LASIK and PRK by numerous clinical reports^7,8,10^, we believe that ray tracing represents the currently best available benchmark for IOL power calculation in post-SMILE eyes.

A plethora of empirically optimized formulas is available in the ASCRS online calculator to accurately predict IOL power after excimer-based keratorefractive surgery (i.e. PRK and LASIK) irrespective of the availability of preoperative refractive data^12^. In our analysis, the Masket formula showed closest agreement with ray tracing in predicting IOL power after SMILE with a ME of −0.36 ± 0.32D and 70% of eyes within ±0.50D. This relatively simple regression formula, originally developed for eyes with prior laser refractive photoablation, modifies the predicted IOL power by taking into account the surgically induced refractive change at the corneal plane^3^. The unpublished Barrett True K formula can be used with or without considering the surgically induced refractive change because it employs an internal regression formula to estimate the surgically induced change in manifest refraction when those data are unavailable^13^. Interestingly, in this sample of post-SMILE eyes, the accuracy of the Barrett True K formula was not clinically significantly deteriorated when preoperative refractive data were not entered but estimated (Barrett True K No History formula). Together with the Potvin-Hill method, these two formulas yielded predictions of refractive error and ±0.50D accuracies statistically non-inferior to the best formula (Masket). The Potvin-Hill method takes into account the corneal true net power calculated for the central 4.0 mm zone derived from Scheimpflug tomography readings, which are subsequently inserted into the Shammas formula^14^. Of note, however, the original Shammas formula^4^ that uses adjusted keratometry readings showed the greatest overestimation of IOL power of all investigated formulas and significantly inferior accuracy as compared with the four aforementioned formulas. With respect to the robustness of IOL power estimation, we were surprised to find less than 5% outliers outside the ±1.00D PE interval for all formulas except the Shammas, the Haigis-L and the Modified Masket formula. All other formulas showed statistically comparable ±1.00D accuracies ranging between 90% and 98%.

The peer-reviewed literature on IOL power prediction after SMILE is surprisingly sparse, considering the increasing popularity of the technique. Two previous studies set out to determine the accuracy of total corneal refractive power measurements in post-SMILE eyes by comparing Pentacam-derived equivalent K readings^15^ and total corneal refractive power measurements^16^ with the clinical history method considered as the golden standard of reference. The latter method, however, is prone to multiple errors (e.g. due to the subjective nature of manifest refraction as well as potential postoperative refractive changes owing to the maturing cataractous lens). Furthermore, no actual IOL power calculation was performed in these studies.

Limitations to the present study may be found. First and foremost, ray tracing was considered as the gold standard method. Even though it has been clearly demonstrated that this technique enables accurate IOL power calculation in treatment-naïve eyes^17,18^ and post-photoablation eyes^7,8,10^, its applicability in post-SMILE eyes is still not ultimately proven. A methodological limitation to the ray tracing method itself is that exact IOL design information such as physical curvatures, thickness and refractive index is required. A further limitation of the present study is that not all formulas currently available in the ASCRS calculator could be included as no Atlas-, Galilei- or OCT-based corneal measurements were available.

To conclude, this study demonstrated good agreement of IOL power calculation after SMILE between ray tracing and formulas that were originally optimized for eyes with prior excimer-based photoablative procedures. Our results suggest that, when using the ASCRS calculator for IOL power calculation after SMILE, the Masket or Barrett True-K formula (when clinical history is available) as well as the Potvin-Hill or Barrett True-K No History formula (when clinical history is unavailable) may provide highest accuracy. Until sufficient data for empirical formula optimization is available for post-SMILE eyes, the authors recommend utilizing these formulas and - if available - to correlate their IOL power predictions with physical ray tracing.

## Materials and Methods

This prospective, cross-sectional study included patients that had undergone SMILE for treatment of myopia and/or myopic astigmatism. All postoperative measurements were performed at least 3 months after SMILE surgery and postoperative contact lens use was considered an exclusion criterion. The herein reported research adhered to the tenets outlined in the Declaration of Helsinki, institutional review board (IRB of the SMILE Eyes Clinic Linz) approval was obtained for all aspects of this study and subjects gave consent to use their data for analysis and publication.

### Surgical procedure

All SMILE procedures were performed by two highly experienced corneal surgeons (S.G.P., M.D.) utilizing the VisuMax 500-kHz femtosecond laser (Carl Zeiss Meditec AG, Jena, Germany). The principles of the SMILE technique have been described in detail previously^19^. In this sample of eyes, a laser spot spacing of 4.5 µm and a laser cut energy of level 32 (corresponding to 160 nanojoules) was employed. The optical zone size was programmed at 6.5 mm and cap thickness ranged between 120–140 µm^20^.

### IOL power calculation

IOL power calculation was performed utilizing dedicated ray tracing software (Okulix, Version 8.81) based on corneal tomography scans (Pentacam HR, Software Version 1.17r139; both: Oculus Optikgeräte GmbH, Wetzlar, Germany), optical biometry and anterior chamber depth measurements (both: IOLMaster 500, Software Version 7.7.7.0330; Carl Zeiss Meditec AG, Jena, Germany). Ray tracing based IOL power calculation was performed for the IOL model CT Asphina 404 (Carl Zeiss Meditec AG) aiming for plano residual refraction. The predicted postoperative residual refractive error of the IOL determined by ray tracing was compared with the residual refraction of the same IOL model and power as predicted by a range of empirically optimized IOL power calculation formulas. The American Society of Cataract and Refractive Surgery (ASCRS) post-keratorefractive surgery IOL power calculator (Version 4.8; http://iolcalc.ascrs.org; last accessed 04/12/2019) was used to calculate the predicted residual refractive error using the following formulas that take into account pre-keratorefractive data: Barrett True K, Masket^3^, Modified Masket. Moreover, the following formulas available in the ASCRS calculator were used, which do not incorporate preoperative data: Barrett True K No History, Haigis-L^5^, Potvin-Hill^14^ and Shammas^4^. As described in detail elsewhere^14^, the Potvin-Hill method uses Pentacam Scheimpflug imaging (Oculus Optikgeräte GmbH, Wetzlar, Germany) to estimate post-LASIK/PRK corneal power from the value “true net power” calculated for the 4.0 mm zone centered on the corneal apex as well as the Shammas formula for IOL power calculation. When appropriate, optimized IOL constants were used as published on the User Group for Laser Interference Biometry (ULIB) website (http://ocusoft.de/ulib/index.htm; last accessed 04/12/2019).

### Subjective manifest refraction

Subjective manifest refraction was measured a minimum of three months after SMILE using the Jackson cross-cylinder method and the standard ETDRS visual acuity chart at 4 m distance.

### Statistical analysis

On the basis of a recent editorial on protocols for studies of IOL formula accuracy^21^, the prediction error (PE) was defined as the difference between the residual refraction predicted by ray tracing and the residual refraction predicted by the respective IOL power calculation formula for the same IOL power and model. The arithmetic mean of the PE was referred to as the mean error (ME). Moreover, all negative errors were converted to positive to calculate the mean absolute error (MAE) as well as the median absolute error (MedAE). Furthermore, the standard deviation, minimum and maximum (range of PE) as well as the percentage of eyes within ±0.25, ±0.50, ±0.75 and ±1.00 diopter (D) were reported^21^. Boxplots were created to illustrate the differences in PE between different IOL power calculation formulas. Analysis of variance (ANOVA) with Tukey post hoc testing was performed to assess the differences in refractive prediction error between formulas. Statistical comparisons between absolute prediction errors were performed using repeated measures analysis of variance (Friedman test with Wilcoxon signed-rank post hoc analyses for nonparametric samples and Bonferroni correction). Fisher’s exact test with Bonferroni correction was employed to compare the proportions of eyes within certain refractive prediction error ranges (±0.50D and ±1.00) as yielded by the respective formulas. A p-value of <0.05 was defined as indicative of statistical significance. All statistical analyses were performed using SPSS 25.0.1 for Windows (IBM Corp.; Armonk, NY, USA).

## Supplementary information

Supplementary Information.
